# Acute respiratory distress syndrome in a child with severe epileptic disorder treated successfully by extracorporeal membrane oxygenation: a case report

**DOI:** 10.1186/s12887-015-0348-1

**Published:** 2015-04-01

**Authors:** Nobuyuki Nosaka, Shingo Ichiba, Kohei Tsukahara, Emily Knaup, Kumiko Hayashi, Shingo Kasahara, Yoshinori Kobayashi, Makio Oka, Katsuhiro Kobayashi, Harumi Yoshinaga, Yoshihito Ujike

**Affiliations:** Advanced Emergency and Critical Care Medical Center, Okayama University Hospital, Okayama, Japan; Department of Pediatrics, Okayama University Graduate School of Medicine, Dentistry and Pharmaceutical Sciences, Okayama, Japan; Department of Community and Emergency Medicine, Okayama University Graduate School of Medicine, Dentistry and Pharmaceutical Sciences, Okayama, Japan; Department of Cardiovascular Surgery, Okayama University Graduate School of Medicine, Dentistry and Pharmaceutical Sciences, Okayama, Japan; Department of Child Neurology, Okayama University Graduate School of Medicine, Dentistry and Pharmaceutical Sciences, Okayama, Japan

**Keywords:** Acute respiratory distress syndrome (ARDS), Extracorporeal membrane oxygenation (ECMO), Severe epileptic disorder, Pediatrics

## Abstract

**Background:**

Extracorporeal membrane oxygenation (ECMO) is now a candidate therapy for children with acute respiratory failure.

**Case presentation:**

We report our experience of using central ECMO therapy for acute respiratory distress syndrome followed by seizure in a 15-month-old girl with a severe epileptic disorder. Her respiratory distress was refractory to standard medical treatment and mechanical ventilatory support. Her condition was complicated by development of a pneumothorax. The patient was successfully weaned off ECMO and discharged without deterioration of her neurological status.

**Conclusion:**

The successful outcome in this case resulted from the central ECMO, which enabled “lung rest” and adequate cerebral blood flow. In skilled ECMO facilities, early implementation of ECMO would give some advantages to patients such as the one presented here. Given the invasiveness and the ease of the procedure, introduction of dual-lumen catheters adequately sized for pediatric patients in Japan is required.

## Background

Extracorporeal membrane oxygenation (ECMO) is now a candidate therapy for patients with acute respiratory failure [1-4] based on several reports of ECMO treatment for acute respiratory failure associated with influenza A H1N1 infection in both adult and pediatric patients [5-10]. Implementation of ECMO for pediatric respiratory failure has almost doubled since 2000. Approximately 350 patients have been treated annually over the past 3 years worldwide, with a 56% survival rate [11]. We report our experience of using ECMO for acute respiratory distress syndrome (ARDS) followed by seizure in a 15-month-old girl with a severe epileptic disorder. Her respiratory distress was refractory to standard medical treatment and mechanical ventilatory support and complicated by development of a pneumothorax. The patient was successfully weaned from ECMO and discharged home without deterioration in her neurological status.

## Case presentation

A 15-month-old girl (10.1 kg, 80 cm) presented to our emergency department because of status epilepticus. She had been diagnosed as having Dravet syndrome [12] with *SCN1A* mutation at the age of 10 months and had received antiepileptic therapy with stiripentol, valproic acid, and clobazam. Psychomotor retardation had been detected at the age of 11 months. She had continued to be subject to repeated convulsions, which were managed by as-needed rectal diazepam.

She was having a partial seizure on arrival at hospital, which was aborted with intravenous midazolam. She was intubated for airway protection and transferred to our emergency intensive care unit. Sulbactam/ampicillin (300 mg/kg/day) was administered for suspected aspiration pneumonia. Once her body temperature had been controlled, she was free of seizures and did not require increased doses of antiepileptic drugs.

Her respiratory function deteriorated, however, and she developed ARDS the day after her admission. Her partial pressure of O_2_ (PaO_2_)/fraction of inspired O_2_ (FIO_2_) (P/F) ratio was 207. She was placed on synchronized intermittent mandatory ventilation. The ventilator settings had to be gradually increased during the next 4 days from 18/5 cmH_2_O to 20/10 cmH_2_O with an increase in FIO_2_ from 0.40 to 0.80. On day 6, the ventilator settings were changed to high-frequency oscillation ventilation (HFOV) because her P/F ratio and oxygenation index (OI) had worsened to 84 and 19, respectively. Having achieved complete muscle relaxation, the HFOV settings were adjusted (mean airway pressure 32 cm H_2_O, FIO_2_ 0.65, stroke volume 60 mL, frequency 10 Hz), after which the P/F ratio and OI improved to 196 and 15.2, respectively.

Her respiratory status stabilized on HFOV. However, on day 8 her oxygenation suddenly deteriorated to a P/F ratio of 54 and OI of 55 because of development of a right pneumothorax (a complication of HFOV), which required urgent placement of a chest tube. Despite a de-aeration procedure, her pulmonary function deteriorated to an OI of 78.9. A decision was made to introduce venovenous ECMO therapy. A 14 Fr return cannula was successfully inserted percutaneously into the left internal jugular vein. However, an attempt to place a 17 Fr drainage cannula into the right internal jugular vein percutaneously failed because of an error in guidewire handling and formation of a hematoma on her neck.

In this emergency situation, the therapeutic strategy was aggressively changed to venoarterial ECMO. With the help of a cardiac surgeon, a 21 Fr drainage cannula and 12 Fr return cannula were placed in the right atrium and ascending aorta, respectively, via mid-sternotomy. The ECMO flow was initially set at 1 L/min with rotational frequency of the centrifugal pump. After introduction of 70% O_2_ via ECMO, her arterial blood gas values dramatically improved (PaO_2_ 404 mmHg), and the FIO_2_ from mechanical ventilation was reduced to 30% with a lower tidal volume and lower airway pressure (positive end-expiratory pressure 8 cmH_2_O, inspiration time 1.5–1.8 seconds, frequency 5/min). Because of continuous oozing from the thoracotomy site and hematoma formation in the right thoracic cavity, substantial volumes of blood were transfused during ECMO management (red blood cell concentrate 3250 mL; fresh frozen plasma 920 mL; platelets 1350 mL). As the patient was already fluid-overloaded because of her severe hypoxic and inflammatory status prior to introducing ECMO, continuous renal replacement therapy was introduced after stabilization on ECMO to control her fluid balance precisely. To reduce the risk of bleeding, nafamostat mesilate was used as an anticoagulant, with a targeted activated clotting time of 150–160 s. The ECMO circuit used had a heparin coating.

**Figure 1:**
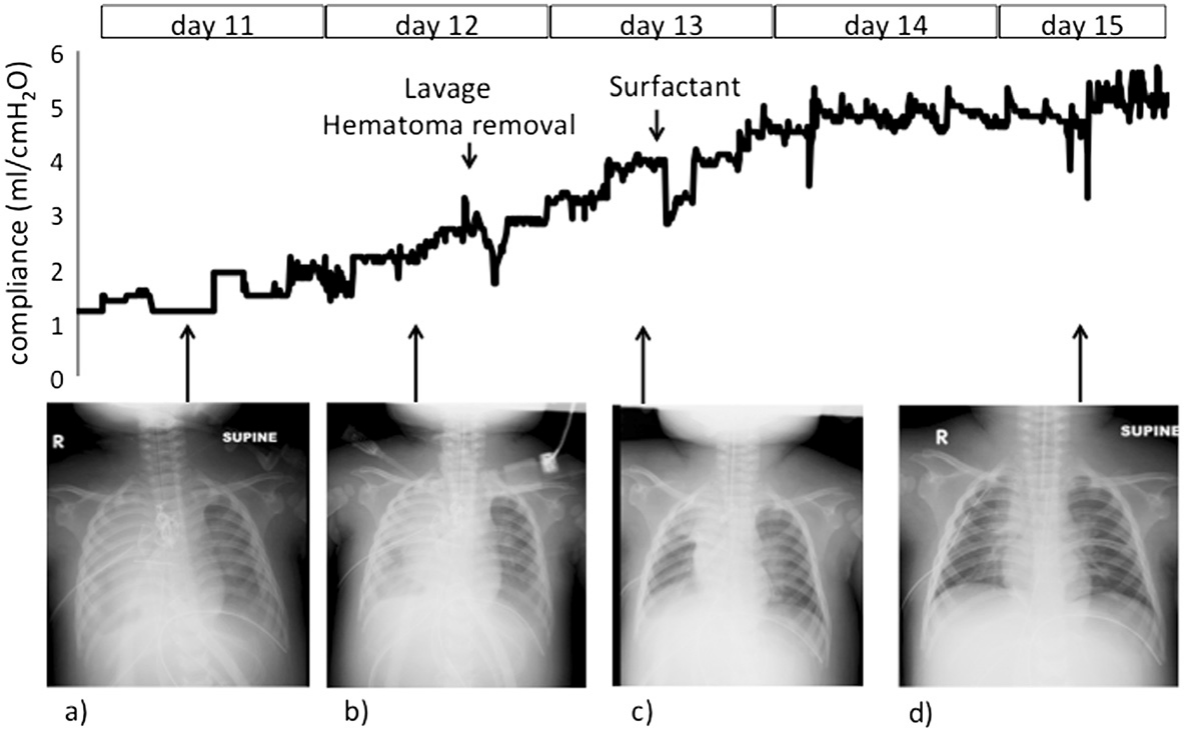
**Lung compliance and chest radiographs.** The line graph shows improvement in lung compliance as calculated by the mechanical ventilator. Lung compliance was temporarily compromised with interventions such as lavage or surfactant administration; however, it subsequently recovered to adequate levels. Parallel with recovery of lung compliance, the radiolucency of the lung gradually improved. **a)** Day 11. **b)** Day 12 before lavage and hematoma removal. **c)** Day 13 before surfactant administration. **d)** Day 15.

On day 12 (fourth day of ECMO), mediastinal lavage and removal of hematoma from the right thoracic cavity was performed through re-thoracotomy with no complications. On day 13 (fifth day of ECMO), 600 mg of surfactant was administered mainly into the right upper and middle lobar branches, with direct bronchoscopic observation. According to her lung compliance measured by the ventilator (Evita Infinity V500, Dräger, Lübeck, Germany) and serial chest X-ray films, there was gradual improvement, especially after the surfactant therapy (Figure 1). During ECMO support, central venous oxygen saturation was stable at around 70%.

Stable hemodynamics having been achieved after 7 days of ECMO support, the patient was successfully weaned from ECMO on day 15. Continuous renal replacement therapy was discontinued simultaneously. Tracheal extubation was performed on day 26. She was discharged from our intensive care unit on day 34 and discharged home on day 79 without deterioration in her neurological status.

Support from multiple departments facilitated rapid introduction of central ECMO and stabilization of the patient, which contributed greatly to successful treatment of this pediatric patient with ARDS [2]. There may be counterarguments against using central respiratory ECMO, but it enabled provision of complete “lung rest,” attenuating ventilator-induced lung injury [13,14], as did aggressive treatment with the surfactant. Because of the patient’s abnormal vessel course and small vessel size, which are associated with Dravet syndrome, there was no adequately sized double-lumen cannula for this patient that was available in Japan. The use of bilateral jugular vein cannulation in ECMO has some risk of increasing the intracranial venous pressure and development of superior vena cava syndrome. Also, if we had chosen to cannulate the jugular veins bilaterally in this patient, it may have caused deterioration in her neurodevelopmental status. Therefore, we believe that central ECMO was a reasonable choice for this patient.

We decided to implement ECMO after detecting a pneumothorax, which is one of the complications of HFOV. Whether the timing of ECMO introduction was optimal in this patient is open to debate. Our respiratory management strategy for pediatric ARDS patients is based on the OI as follows: With OI ≥20, implement HFOV; with OI ≥30, implement ECMO. Two large randomized controlled trials comparing HFOV with conventional lung-protective ventilation were published in 2013: the Oscillation for Acute Respiratory Distress Syndrome Treated Early (OSCILLATE) trial [15] and the Oscillation in ARDS (OSCAR) trial [16]. These trials suggested that, in adults with early moderate-to-severe ARDS, HFOV conferred no benefit and may have been harmful [17]. Historically, HFOV is preferred for lung protective therapy in small children. However the pathophysiology of acute respiratory failure in neonates is known to differ from that of older infants and children. Thus, HFOV may also be harmful to children. With sufficient understanding of the characteristics of both HFOV and ECMO, lowering the criteria for implementation of ECMO might improve patients’ safety and outcomes.

Easier, less invasive implementation of ECMO should provide great advantages for pediatric patients with severe ARDS. The current technique in Japan involves implementing venovenous ECMO using bilateral jugular veins or venoarterial ECMO using an internal jugular vein and a common carotid artery. The internal diameter of the catheter is crucial to facilitating sufficient drainage of venous blood [18]. However, the available catheters with sufficiently large diameters (larger than 15 Fr) in Japan may be too long for pediatric patients. This makes aspects of the cannulation procedure (e.g., handling of the guidewire) more difficult, as we experienced in this patient. Recently, dual-lumen catheters have gained popularity in western countries for administering pediatric ECMO [19]. Currently, these devices are unavailable in Japan. However, given the invasiveness and the ease of the procedure, introduction of dual-lumen catheters for pediatric patients is urgently required in our country.

ECMO enabled safe administration of surfactant into targeted lung lobar branches with bronchoscopic guidance. ECMO provides a safe setting for procedures that are risky during ventilation. Several researchers have reported that surfactant is effective in patients with ARDS, especially children [20,21]. Based on our observations of improvement in lung compliance, we believe that surfactant contributed to our patient’s recovery. However, further research to verify the effect of surfactant in ARDS patients is needed. It would be desirable to accumulate a series of pediatric ARDS patients who have been treated with surfactant and in whom relevant variables, including lung compliance, have been measured.

Severe neurological disorders have been thought to contraindicate the use of ECMO [22]. According to the most recent Extracorporeal Life Support Organization guidelines, however, most contraindications are relative. Hence, the risk of a procedure should be balanced against its potential benefits [18]. Pneumonia is one of the most frequent causes of death in epileptic patients, including those with Dravet syndrome [23-25]. The development of ECMO has increased the range of means of managing such patients. However, ECMO remains an expensive treatment with a high risk of complications. The indications should be carefully scrutinized, case by case. From this perspective, the possibility of implementing ECMO should be considered at an early stage to ensure sufficient time to discuss its applicability.

## Conclusions

We implemented central venoarterial ECMO for respiratory failure in a pediatric patient with a severe epileptic disorder in an emergency situation. Central respiratory ECMO could be a reasonable choice for small pediatric patients with severe ARDS. With enough understanding of the characteristics of ECMO, its early implementation might improve outcomes. Our results suggest that the introduction of dual-lumen catheters for pediatric patients to Japan could lead to safer, less invasive implementation of ECMO.

### Consent

Written informed consent was obtained from the parents of this patient for publication of these data and any accompanying images. A copy of the written consent is available for review by the Editor of this Journal.

## Abbreviations

ARDS: Acute respiratory distress syndrome
ECMO: Extracorporeal membrane oxygenation
FIO_2_: Fraction of inspired O_2_
HFOV: High-frequency oscillation ventilation
OI: Oxygenation index
PaO_2_: Partial pressure of oxygen
P/F: Pressure of O_2_/fraction of inspired O_2_
